# The learning curve in short-stem THA: influence of the surgeon’s experience on intraoperative adjustments due to intraoperative radiography

**DOI:** 10.1007/s00590-017-2049-y

**Published:** 2017-10-13

**Authors:** Lennard Loweg, Karl Philipp Kutzner, Matthias Trost, Marlene Hechtner, Philipp Drees, Joachim Pfeil, Michael Schneider

**Affiliations:** 1 Department for Orthopaedic Surgery and Traumatology, St. Josefs Hospital Wiesbaden, Beethovenstr. 20, 65189 Wiesbaden, Germany; 2 Department for Orthopaedic Surgery and Traumatology, St. Josef-Hospital, University Bochum, Bochum, Germany; 3 Institute of Medical Biostatistics, Epidemiology and Informatics (IMBEI), University Medical Center of the Johannes Gutenberg University Mainz, Mainz, Germany; 4 Department for Orthopaedics and Traumatology, University Medical Center of the Johannes Gutenberg University Mainz, Mainz, Germany

**Keywords:** Total hip arthroplasty, Short stem, Intraoperative radiography, Learning curve, Adjustments, Optimys

## Abstract

**Introduction:**

Short-stem THA has become increasingly popular over the last decade. However, implantation technique differs from conventional THA and thus possibly involves a distinct learning curve. The purpose of this study was to evaluate the value of intraoperative radiography and the influence of the surgeon’s experience on intraoperative adjustments in short-stem THA.

**Methods:**

A total of 287 consecutive short-stem THAs, operated by a total of 24 senior consultants, consultants and residents in training, were prospectively included. Intraoperative radiography was performed after trial reduction. Preoperative planning and intraoperative outcome with regard to positioning, sizing of components as well as resulting offset and leg length were compared. Frequency, reason and type of intraoperative adjustments were documented in relation to the surgeon’s experience. Operation time was assessed.

**Results:**

One hundred and fifty-six (54.4%) procedures were carried out by one of three senior consultants, and a total of nine consultants and 12 residents in training performed 105 (36.6%) and 26 (9.0%) operations, respectively. In 121 cases (42.2%), intraoperative adjustments were made following intraoperative radiography. Intraoperative adjustments of one or more components were made by senior consultants in 51 cases (32.7%), by consultants in 53 cases (50.5%) and by residents in 17 cases (65.4%), respectively. The most common cause was undersizing of the stem. Operation time varied markedly between groups of surgeons.

**Discussion:**

Short-stem THA involves a learning curve. Intraoperative radiography is decisive for prevention of malpositioning and undersizing of components, as well as loss of offset and leg length discrepancies. Hence, it should be considered mandatory, especially for less experienced surgeons.

## Introduction

In the course of new developments in total hip arthroplasty (THA) over the last decade, short stems became increasingly popular [1, 2]. Compared to conventional straight stems, new designs aim at conserving proximal bone stock and at allowing soft tissue sparing implantation [3, 4]. In modern THA, the reconstruction of the individual anatomy of the hip joint is of great importance [5]. An adequate restoration of the physiological femoro-acetabular offset and leg length as well as a desired intraoperative correction of anatomical variants or pathologies is crucial in precise positioning of components [6].

Due to the curved design in calcar-guided short stems, an individual stem positioning is possible in a wide range of different varus and valgus alignments alongside the medial calcar [7]. However, the implantation technique differs from conventional THA and involves a learning curve. Consequently, especially young and inexperienced surgeons might face challenges regarding sizing and positioning of short stems adequately.

Preoperative planning of implant positioning and implant sizing, using a digital 2D templating software, is today considered mandatory in THA [8, 9]. It allows a distinct analysis of the individual preoperative anatomy and makes it possible to recognize and address possible intraoperative pitfalls before surgery. Malpositioning, undersizing, leg length discrepancies or undesired offset changes could possibly be avoided [9].

In order to intraoperatively compare the preoperative planning with the result obtained after inserting the trial implant, intraoperative radiography can be performed using an image intensifier [10]. In conventional THA, results of studies assessing the value of intraoperative radiography are inconsistent. While it was found to be a useful method for detecting errors of placing the femoral components [11], other results led to a discouragement of the usage in uncomplicated primary THA due to increased operative time and associated costs [12]. Consequently, the use of intraoperative radiography is still underappreciated and regularly waived by many surgeons.

The aim of the present prospective study was to determine frequency, reasons and types of intraoperative adjustments after performing intraoperative radiography in calcar-guided short-stem THA in relation to the surgeon’s experience.

Our hypothesis was that in calcar-guided short-stem THA the usage of intraoperative radiography is crucial for correct implant positioning and sizing and should be considered mandatory.

## Materials and methods

In the present prospective observational study, 287 consecutive patients receiving short-stem THA were included from February to June 2016 at a single institution, operated by a total of 24 different surgeons and residents in training. All patients previously gave written consent to participate in the study. Approval was obtained from the local review board prior to inclusion.

Preoperatively, a standard antero-posterior radiograph and an axial view were prepared using a 30-mm planning ball to ensure accurate scaling. Preoperative planning was carried out using mediCAD Classic software (version 3.50.0.1, Hectec, Landstuhl, Germany), and all operations were performed accordingly.

In all patients, the investigated calcar-guided short-stem optimys (Mathys Ltd., Bettlach, Switzerland) was implanted. It is a femoral neck, partially preserving prosthesis made of titanium alloy, which is available in 12 different sizes with a 12/14 mm cone and two different offset versions. The lateral version increases the offset by 5 mm without changing the leg length. The stem is aligned along the proximal medial cortex and the calcar. Anchoring is based on the fit-and-fill principle and can be done as three-point anchoring in some cases. The triple conical shape aims to obtain good primary stability and prevent migration. The greater trochanter region remains intact.

In majority of the cases, the stem was combined with a cementless monoblock cup (RM Pressfit vitamys, Mathys Ltd., Bettlach, Switzerland). In 15 cases (5.2%) a cementless modular cup was used (Fitmore, Zimmer, Indiana, USA). Only ceramic heads with a diameter of 28 or 32 mm, according to the diameter of the cup, were used in three different head lengths (S, M, L).

In all surgeries, a minimally invasive, modified anterolateral approach in supine position was used [13].

In all cases, intraoperative radiography was performed after the implantation of the definite acetabular component within the scope of trial reduction using the trial rasp, trial neck and trial head. At least one anterior–posterior (AP) and one axial radiograph of the hip joint were produced using a sterile-covered image intensifier (Arcadis Varic, Siemens, Munich, Germany).

**Fig. 1:**
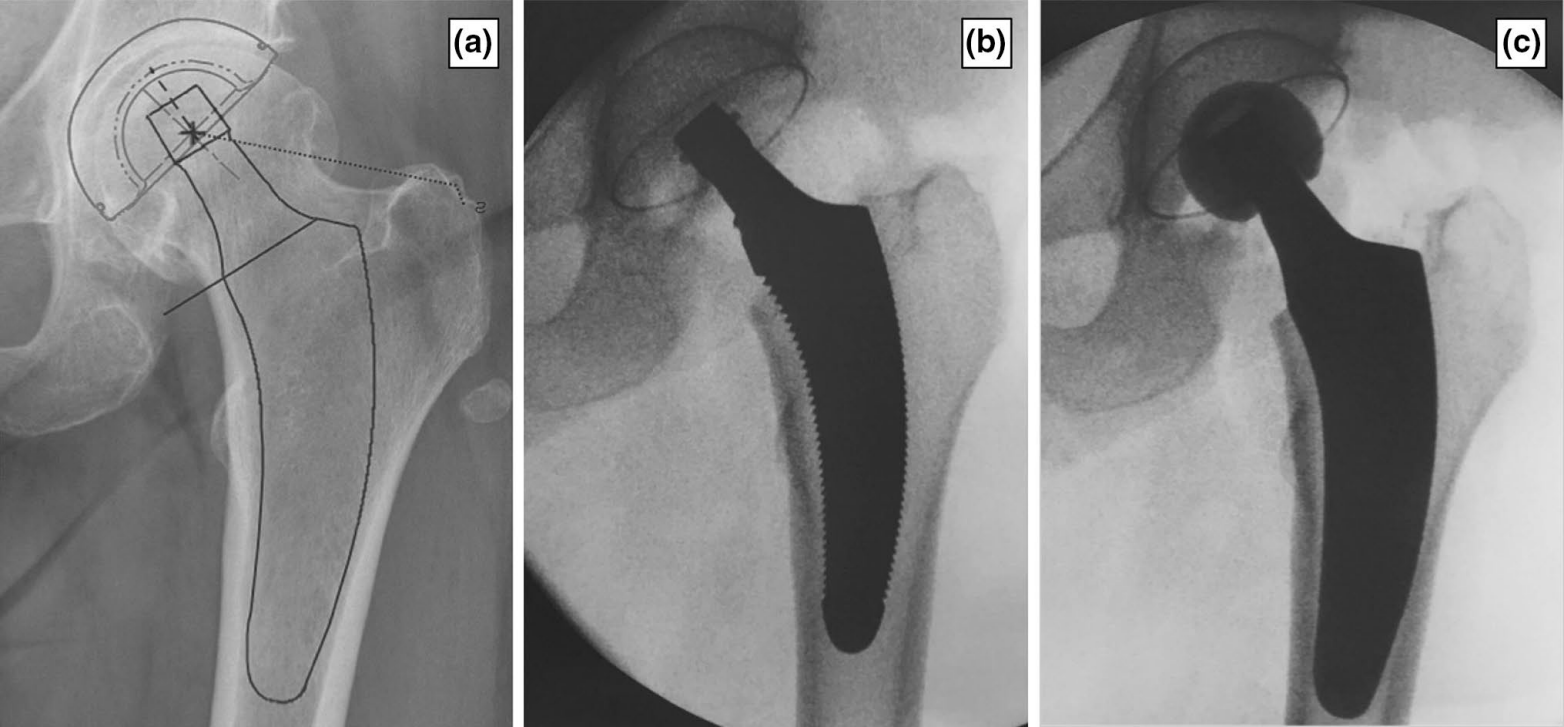
**a** Preoperative planning. **b** Intraoperative radiography with the trial implant of the planned size. Compared to the preoperative planning, it appears to be undersized and misses lateral cortical contact. **c** Radiography with the original implant after upsizing. The result shows adequate positioning matching the preoperative planning

The positioning and sizing of components as well as the resulting femoro-acetabular offset and leg length were assessed, using landmarks such as the lesser trochanter and intraoperatively compared to the preoperative planning. In case of malpositioning and undersizing of components or inadequate offset and leg length, adjustments were made accordingly. Following final reduction, visualization of final results in two planes was repeated (Fig. 1a–c).

It was documented whether the regularly performed intraoperative radiography explicitly led to intraoperative adjustments for one of the components used. The corresponding reasons and the types of adjustment were assessed. Furthermore, the surgeon’s experience (senior consultant, consultant or resident) and duration of procedure were documented.

Primary outcome was the frequency of intraoperative adjustments. Secondary outcomes were reason and type of the adjustments as well as the procedure duration.

### Statistics

Categorical data were described by absolute and relative frequencies. Continuous variables were described by mean, standard deviation (SD), median and range. Chi-square test was executed to compare, if an influence of the surgeon’s experience could be detected on the frequency of intraoperatively performed changes [14]. Further analyses were regarded as explorative without adjustment for multiple testing. An ANOVA was used to compare the duration of procedure between the groups. A *p* value < 0.05 was considered to be indicative of statistical relevance. SCOPE vs 2.0 (numerics data GmbH, Zurich, Switzerland) was used for the calculations.

### Ethics

This study was conducted in accordance with the 2013 version of the Declaration of Helsinki. The study protocol was approved by the Freiburg Ethics Commission International. A positive vote was dated 10/04/2010 (feci Code: 010/2071). No patient was enrolled until written agreement from the ethics committee was obtained.

## Results

A total of 287 procedures were performed by a total of three highly experienced senior consultants, nine experienced consultants and 12 residents in training with a senior consultant or consultant as first assistant within the scope of a certified hospital for high-volume endoprosthetic surgery. One hundred and fifty-six (54.4%) of the surgeries were carried out by one of the senior consultants, and the consultants and residents performed 105 (36.6%) and 26 (9.0%) operations, respectively.

**Fig. 2:**
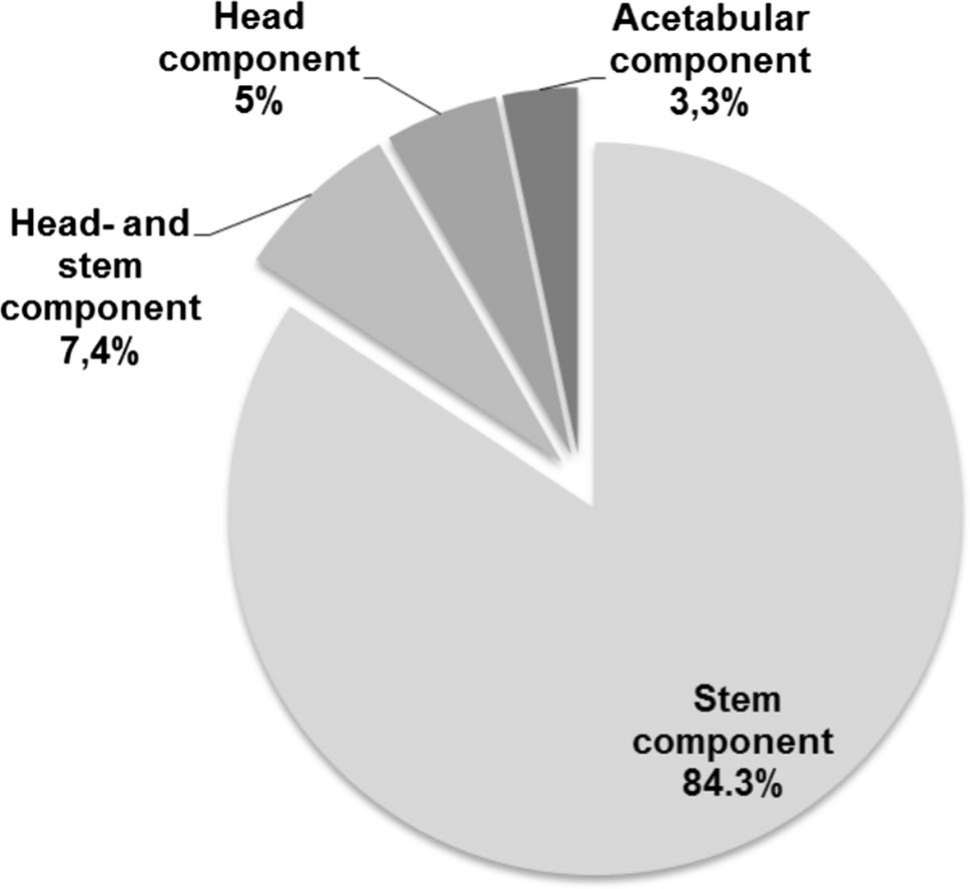
Percentage of intraoperative adjustments based on components after performing intraoperative radiography

In 121 cases out of 287 procedures (42.2%), intraoperative adjustments were made following intraoperative radiography. In 102 of the 121 documented changes (84.3%), only the stem component was modified. In 9 cases, changes to the stem and head component were made while in 6 cases, changes to the head component were necessary. In four cases (1.4%), a repositioning of the already implanted original acetabular component resulted following intraoperative radiography (Fig. 2).

**Fig. 3:**
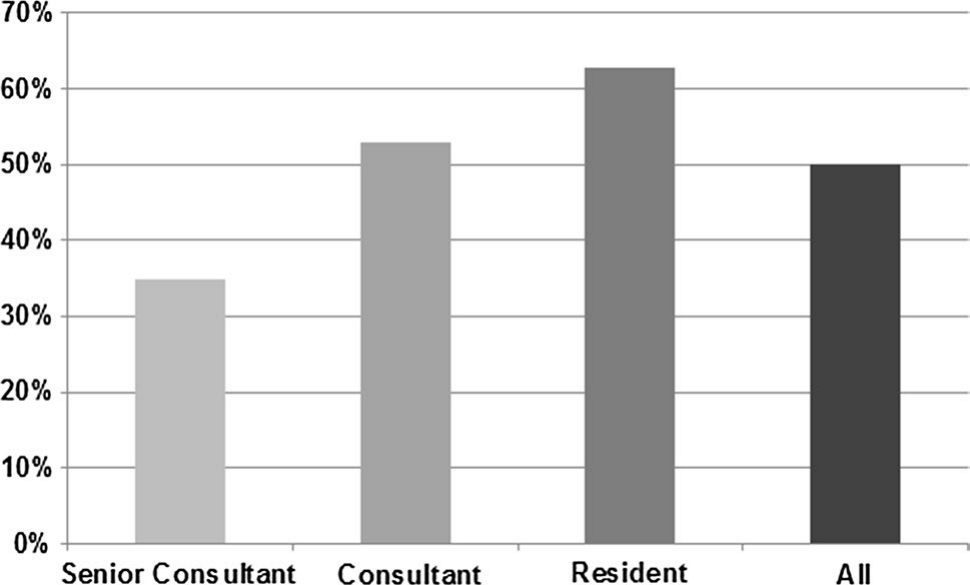
Percentage of intraoperative adjustments made following intraoperative radiography for the three groups of surgeons providing different levels of experience in short-stem THA

Frequencies of intraoperative adjustments varied significantly between groups of senior consultants, consultants and residents. While intraoperative adjustments of one or more components were made by the senior consultants in 51 cases (32.7%), consultants and residents had to adjust components in 53 cases (50.5%) and 17 cases (65.4%), respectively (*p* < 0.001) (Fig. 3).

A total of nine different reasons for the necessity of intraoperative adjustments of one of the components were documented. Undersizing of femoral trial rasp accompanied with missing contact to the lateral cortical bone was identified as the most common cause (26.1%) (Fig. 1b). The exact details of all reasons and types of adjustments are shown in Table 1. There were no differences regarding reasons and types of adjustments between the three groups of surgeons.

**Table 1:** Frequency, reasons and types of various adjustments after intraoperative radiography

Reason for adjustment	Frequency of adjustments		Type of adjustment
	*n*	Percent	
Undersized stem	75	62.0	Change to a larger stem
Insufficient offset	13	10.7	Change to a lateral stem
Undersized stem and insufficient offset	10	8.3	Change to a larger stem and lateral offset
Undersized stem and short leg length/instability	9	7.4	Change to a larger stem and larger head
Short leg length/instability	6	5.0	Change to a larger head
Cup position	4	3.3	Adjustment of cup position
Stem too deep	2	1.7	Change to a larger stem
Oversized stem	1	0.8	Change to a smaller stem
Excessive offset	1	0.8	Change to a standard offset

Mean procedure duration varied between the three groups of surgeons. While the senior consultants performed the surgeries in a mean time of 34.7 min (SD 9.7 min; range 19–59 min), the groups of consultants and residents took a mean time of 52.9 min (SD 14.6 min; range 26–87 min) and 62.7 min (SD 11.7 min; range 43–82 min), respectively (Table 2).

**Table 2:** Number of operations and procedure duration for all three groups of surgeons

	Operations (*n*)	Op time mean (min)	Median (min)	SD	Min	Max
All	287	50.1	50.5	16.7	19	87
Senior consultants	156	34.7	32	9.7	19	59
Consultants	105	52.9	51.5	14.6	26	87
Residents	26	62.7	62	11.7	43	82

## Discussion

As part of implantation of a calcar-guided short stem, intraoperative radiography was performed following the trial reposition with the trial rasp, trial cone and trial head in order to compare the preoperative planning with the intraoperative result regarding component sizing, positioning as well as reconstruction of offset and leg length. The importance of intraoperative radiography for the correct sizing and precise positioning of implants could be demonstrated especially for young and less experienced surgeons, although still today surgeons often waive this procedure in clinical practice.

In the current literature, only few studies investigated the value of intraoperative radiography in THA, especially with emphasis on the femoral component [10, 15].

Hofmann et al. [15] described in a retrospective study with 86 patients, a significant reduction regarding risk of postoperative leg length discrepancies due to the usage of intraoperative radiography in conventional THA. It was possible to avoid leg length discrepancies of more than 6 mm. Ezzet et al. showed that a single intraoperative AP-pelvic radiograph could provide information about the position of the acetabular component, alignment of the femoral component and leg length in 200 conventional THAs. They concluded that intraoperative radiography is a fast and easy method in identifying intraoperative misalignments [10], which they could identify and rectify in all cases.

These results were consistent with the results of the present study. However, in calcar-guided short-stem THA, due to a different implantation technique compared to conventional THA, it might even be more important to intraoperatively ascertain visual feedback on stem positioning and sizing.

In the present study, in 42.2% of all cases, adjustments to one or more components were necessary following intraoperative radiography. In over 80% of those cases, the femoral component was adjusted. Calcar-guided short stems have been developed in order to optimally adapt to the anatomy of the proximal femur and to allow a restoration of hip biomechanics [5, 16]. They are aligned alongside the medial cortical bone, sparing the greater trochanter completely [4]. Implantation is done in a “round-the-corner” technique, using individualized levels of osteotomy in order to align the stem in varus- or valgus position, according to the patient’s anatomy [4]. This results in a broad range of CCD angles to be reconstructed with these types of stems [2, 7]. The implantation of a calcar-guided short stem, consequently, can be considered as less standardized but often more individualized compared to diaphyseal anchoring in conventional straight stems. The intraoperative implementation of the preoperative planning is correspondingly important in order to avoid undesired offset changes or leg length discrepancies [5].

Deviations affecting femoral offset and leg length were detected and adjusted following intraoperative radiography in 32.2% of all cases by choosing a different offset version or head size. Adequate reconstruction of offset and a balanced leg length contribute markedly to an excellent joint function and postoperative patient satisfaction [17, 18]. For the investigated stem for each implant, a standard and a lateral offset version were available. The choice of the offset version after intraoperative radiography has an impact on the restoration of the precise anatomy of the patient. Possible loss of function can thus be prevented.

Furthermore, due to a pronounced metaphyseal anchoring mechanism of short stems, the achievement of a stable contact to the lateral cortical bone in the diaphysis, in addition to the metaphyseal ring fixation and the cortical support on the calcar, is critical for initial implant stability [7]. This suggests that the choice of stem size plays a decisive role in achieving primary stability. Using intraoperative radiography allows the detection of the missing cortical contact due to undersizing.

In summary, in calcar-guided short-stem THA frequent intraoperative adjustment of the stem positioning or sizing can be expected after radiography.

From the three groups of surgeons with different levels of experience, higher rates of intraoperative adjustments of at least one component were noted for young and less experienced colleagues (Fig. 3). Given a less standardized implantation technique in short stems, a marked learning curve has to be considered. Even though the residents in training were accompanied by either a consultant or a senior consultant, the frequency of intraoperative adjustments following intraoperative radiography was significantly higher compared to the group of senior consultants and consultants. Advanced experience in short-stem THA reduces the necessity of intraoperative adjustments. However, even the group of highly experienced senior surgeons showed a high rate of adjustments of 32.7%.

Most alterations were related to the femoral component. However, in four cases inclination of the cup was detected to be too steep, resulting in repositioning of the already implanted original acetabular component. Thus, also inadequate positioning of the cup will be detected and can be revised immediately. Further revision due to unforeseen can thus be avoided.

Intraoperative radiography in THA in clinical practice does not seem to be regularly implemented by surgeons around the world, probably owing to time constraints. One of the main reasons might be a feared time loss during surgery. An increase in procedure duration influences demonstrable perioperative risks and costs [19, 20]. However, this investigation shows that a mean operation time of 50 min, including intraoperative radiography, is achievable. Even the group of less experienced residents in training was able to perform short-stem THA in a mean time of 62.7 min. From our experience, intraoperative radiography in supine position is associated with an insignificant time loss of 1–2 min. The differences in operation time between the groups most likely are not due to the usage of intraoperative radiography, but reflect a higher routine in short-stem THA. A risk of increased rates of perioperative infection therefore is not likely to be related to a prolongation of operation time. However, the additional usage of an image intensifier in the operation room potentially involves the risk of affecting sterility.

Another reason for the rejection of intraoperative radiography might be the exposure to radiation for the operating staff. However, by wearing lead gowns and by maintaining the greatest possible distance from the radiation source, a possible radiation exposure can be minimized, both for the surgeon and the surgical staff [21].

There are certain limitations to the present study. First of all, since no randomized study design has been chosen, further confounders have to be considered regarding the influence of the surgeon’s experience. Potentially more difficult cases have been operated by the senior consultants, compared to the residents, leading to a possible bias. Secondly, only one type of short stem was used. Further investigations should address different stem designs as well. Additionally, groups of surgeons providing different levels of experience were not equally sized, thus limiting the validity of the statistical analysis. However, we consider our results to be generally applicable to high-volume orthopedic hospitals.

## Conclusions

Intraoperative radiography is decisive for sizing and positioning of the femoral components in short-stem THA, as it allows a comparison to the preoperative planning during surgery and helps to prevent malpositioning, undersizing as well as undesired loss of offset and leg length discrepancies. Given a less standardized implantation technique in short-stem THA compared to conventional THA, a distinct learning curve has to be considered. Especially for less experienced surgeons, intraoperative radiography has been proven to be inevitable in verification of precise implantation of all components and hence should be considered mandatory. A short procedure duration, including the usage of intraoperative radiography, is achievable with a well-trained staff and standardized methods.
